# Supplementary material for: Bodily ownership of an independent supernumerary limb: an exploratory study
Source: Sci Rep. 2022 Feb 14;12:2339. doi: 10.1038/s41598-022-06040-x (PMC8844351; doi:10.1038/s41598-022-06040-x)
Supplement: Supplementary file 1 — Supplementary Information 1. [file 41598_2022_6040_MOESM1_ESM.pdf]

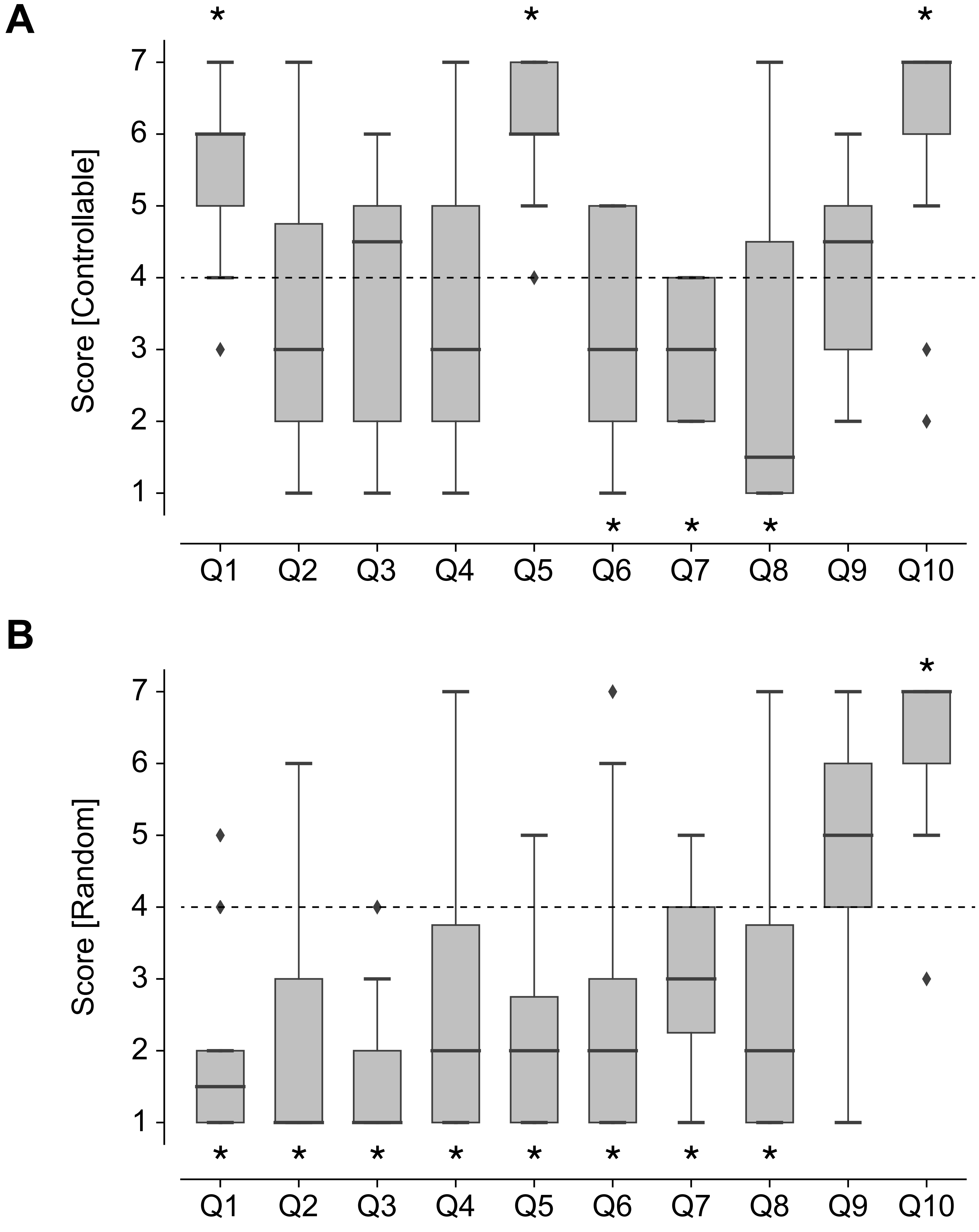

Supplementary figure 1: Results of subjective ratings of questionnaires for A) the controllable condition and B) the random condition. The participants answered seven embodiment-related questions and three control questions on a seven 7-point Likert scale. In each box plot, the thick line indicates the median, and the bottom and the top lines of the box show the first and the third quartiles, respectively. The whiskers extend to the minimum and maximum points of the data or to the points of 1.5 times the interquartile range if the minimum and maximum points exceed them. The diamond markers showed the data points considered as outliers that exceed the whisker range. Asterisks above the boxes indicate statistical significance higher than the medium level (4), whereas those below indicate lower (FDR-corrected $p < 0.05$ for multiple comparisons, the Wilcoxon signed-rank two-sided test).
