# Supplementary material for: Bodily ownership of an independent supernumerary limb: an exploratory study
Source: Sci Rep. 2022 Feb 14;12:2339. doi: 10.1038/s41598-022-06040-x (PMC8844351; doi:10.1038/s41598-022-06040-x)
Supplement: Supplementary file 2 — Supplementary Information 2. [file 41598_2022_6040_MOESM2_ESM.docx]

Supplementary movie 1: The robotic sixth finger in motion. The movie demonstrates the robotic sixth finger (white-colored plastic parts strapped onto the hand near to the left little finger) can be controlled independently of, and simultaneously if required, other innate fingers using EMG signals measured from the forearm by the wireless EMG sensors (black-colored units attached above wrist/finger muscles, covered by a bandage to ensure fixation). The movie was taken by Daichi Ueda.
